# Resistance and resilience of pelagic and littoral fishes to drought in the San Francisco Estuary

**DOI:** 10.1002/eap.2243

**Published:** 2021-01-22

**Authors:** Brian Mahardja, Vanessa Tobias, Shruti Khanna, Lara Mitchell, Peggy Lehman, Ted Sommer, Larry Brown, Steve Culberson, J. Louise Conrad

**Affiliations:** ^1^ United States Bureau of Reclamation 801 I Street, Suite 140 Sacramento California 95814 USA; ^2^ United States Fish and Wildlife Service 850 South Guild Avenue Lodi California 95240 USA; ^3^ California Department of Fish and Wildlife 2109 Arch‐Airport Road Stockton California 95206 USA; ^4^ California Department of Water Resources 3500 Industrial Boulevard West Sacramento California 95691 USA; ^5^ United States Geological Survey 6000 J Street Sacramento California 95819 USA; ^6^ Delta Stewardship Council 980 9th Street Sacramento California 95814 USA

**Keywords:** Chinook salmon, climate variability, delta smelt, drought, estuary, extreme events, fish, largemouth bass, resilience, resistance

## Abstract

Many estuarine ecosystems and the fish communities that inhabit them have undergone substantial changes in the past several decades, largely due to multiple interacting stressors that are often of anthropogenic origin. Few are more impactful than droughts, which are predicted to increase in both frequency and severity with climate change. In this study, we examined over five decades of fish monitoring data from the San Francisco Estuary, California, USA, to evaluate the resistance and resilience of fish communities to disturbance from prolonged drought events. High resistance was defined by the lack of decline in species occurrence from a wet to a subsequent drought period, while high resilience was defined by the increase in species occurrence from a drought to a subsequent wet period. We found some unifying themes connecting the multiple drought events over the 50‐yr period. Pelagic fishes consistently declined during droughts (low resistance), but exhibit a considerable amount of resiliency and often rebound in the subsequent wet years. However, full recovery does not occur in all wet years following droughts, leading to permanently lower baseline numbers for some pelagic fishes over time. In contrast, littoral fishes seem to be more resistant to drought and may even increase in occurrence during dry years. Based on the consistent detrimental effects of drought on pelagic fishes within the San Francisco Estuary and the inability of these fish populations to recover in some years, we conclude that freshwater flow remains a crucial but not sufficient management tool for the conservation of estuarine biodiversity.

## Introduction

Climate change models forecast increased frequency and intensity of drought both in the western United States and globally (Cayan et al. 2010, Williams et al. 2020). Droughts have broad‐scale effects on aquatic ecosystems, including changes to the physical environment and biological communities (Bogan et al. 2015, Dittmann et al. 2015). Whereas changes in abiotic parameters, such as rising temperatures and increasingly variable magnitude and timing of precipitation, are well studied (Easterling et al. 2000, Cloern et al. 2011, Dettinger and Cayan 2014, Dettinger et al. 2016), changes to species abundances and assemblages have received relatively less attention. For example, drought conditions can provide opportunities for invasive species to become established in a new system, with cascading effects on communities even after drought conditions recede (Bêche et al. 2009, Ramírez et al. 2018). This potential for broad community‐level changes is important because the species composition of communities affects the food web and other aspects of ecosystem function. Species population trends are also critical for natural resource managers, who depend on this information to balance ecosystem needs with cultural and economic demands. Population trends become increasingly important for managers under a changing climate regime.

As the frequency of drought increases, an emerging question is whether native ecosystems are resilient or resistant to drought. Ecological theory has defined resilience in multiple ways. Holling (1973) first defined resilience as the ability of ecological relationships to persist in the face of disturbance. Ecologists have also examined resilience in the context of regime shift theory (Scheffer et al. 2001), acknowledging that ecosystems may have multiple or alternative stable states. In this context, resilience is the capacity of an ecosystem to absorb disturbance before shifting to an alternative stable state (Gunderson 2000). Climatic stressors, particularly when combined with other anthropogenic stressors (e.g., pollution, habitat loss), may erode ecosystem resilience (Folke et al. 2004).

It is difficult to measure indicators of ecological resilience when referencing large‐scale ecosystem states. In order to address ecosystem management under a changing climate, it is important to understand and quantify changes in resilience. To measure resilience, the specific concept of “engineering resilience” has been useful. Engineering resilience is defined as the ability of an ecosystem parameter to return to a reference, or pre‐disturbance state (Holling 1996, van Nes and Scheffer 2007), and it can be quantitatively evaluated if the parameter of interest is measured before, during, and after a disturbance event. Recent studies of species' resilience in response to drought cycles have adopted this definition of resilience for the purpose of analysis (Bêche et al. 2009, van Ruijven and Berendse 2010, Bennett et al. 2014), reflecting the ability of species to recover from drought after experiencing its impact.

As “resilience” reflects recovery, “resistance” reflects persistence despite a disturbance and is a different metric of the ecological effects of stressors. Resistance to drought is a species' or community's ability to persist during drought conditions (Bennett et al. 2014, Mac Nally et al. 2014, Selwood et al. 2015). Therefore, measures of resistance evaluate the change in a population during the stressor compared to levels just before the stressor began (Harrison 1979). Species and communities may resist drought by relocating to refugia that offer suitable habitat conditions while a drought continues (Lake 2003, Magoulick and Kobza 2003). However, intrinsic traits of life history and ranges of physiological tolerance are also important predictors of species' capacity to persist despite drought. In a trait analysis of freshwater fish species in Australia's Murray‐Darling Basin, Chessman (2013) illustrated that species with low fecundity, lower thermal limits, and more restricted diets have lower resistance to drought. In droughts of exceptional severity that are supplemented with other stress factors (e.g., high temperatures), however, species traits may not be related to resistance (Bennett et al. 2014). Regardless of the mechanism for resisting drought, species that have high resistance may have low resilience, simply because they are maintaining their populations through drought and wet conditions. To understand the capacity for species and communities to cope with increased frequency and severity of drought, both resistance and resilience must be evaluated.

Among ecosystems that may be significantly altered by climate change, estuaries represent a distinctive and extremely dynamic ecosystem at the interface of ocean and terrestrial habitats (Kennish 2002, Ray 2005, Cloern et al. 2017). Estuaries are also societally important systems, offering a suite of ecosystem services including transport, water supply, recreation, water quality improvements, and wildlife habitat (Barbier et al. 2011). However, many of these services depend upon the resilience and resistance of the ecosystem to an array of stressors, including contaminants, habitat modification and loss, as well as invasive species. Increased frequency and severity of drought are major stressors to estuarine ecosystems. Ecosystem resilience to drought impacts may be increasingly compromised such that estuarine biological communities are vulnerable to lasting changes after droughts (Wetz and Yoskowitz 2013, Kimmerer et al. 2019). Measuring such effects can be difficult in the absence of quantifiable metrics. Estuaries typically support a robust resident fish community in addition to serving as migration routes, refugia, and nursery grounds for a number of other fishes (Elliott et al. 2007). For these reasons, fish communities have often been used as indicators for the health and ecosystem resilience of the estuaries (Harrison and Whitfield 2004).

Evaluating and quantifying species resilience to drought requires long‐term data sets to examine changes over successive drought cycles. Given the typical variability in the frequency and duration of drought and wet cycles, data sets used for analyzing resilience must span multiple decades. Long‐term monitoring programs that collect data on multiple species in a consistent manner for multiple decades are uncommon. The fish monitoring programs of the San Francisco Estuary (SFE) of California have been conducted continuously for up to five decades and provide an opportunity to study species and community data over multiple drought cycles. The SFE has experienced major ecological change, including the decline of a suite of pelagic fishes (Mac Nally et al. 2010, Thomson et al. 2010). The system is also highly invaded by nonnative species (Cohen and Carlton 1998), including bivalves that have brought about major changes to the food web by significantly increasing the grazing pressure on plankton communities (Kimmerer et al. 1994, Kimmerer 2006). Significant, multi‐year droughts occur in the SFE and its watershed about every fifteen years (Dettinger and Cayan 2014). Some ecological effects of drought have already been characterized, and include increased harmful algal blooms (Lehman et al. 2017), possible facilitation and establishment of invasive species (Winder and Jassby 2011, Kimmerer et al. 2019), changes in water residence time and primary productivity (Glibert et al. 2014), and increased contaminant exposure to fishes (Bennett et al. 1995). The SFE system illustrates the issue of multiple stressors impacting estuaries, including climate‐related changes, through consistent fish monitoring since 1967.

In this paper, we leveraged two long‐term data sets to investigate the capacity of a suite of fish species in the SFE to exhibit resistance and resilience to periodic drought over five decades. We focused on fish species with 1‐ or 2‐yr life cycles and young‐of‐years, as we expect them to exhibit more immediate response to droughts. Specifically, our three study questions are as follows: (1) How do we define drought cycles from 1967 to 2017 in the SFE? (2) Do fish species exhibit drought resistance? (3) Do fish species recover from drought when wet conditions return (i.e., resilience)? The fishes of the SFE watershed and estuary have evolved in a Mediterranean climate characterized by periodic floods and droughts and have life histories with high resistance or resilience to drought (Moyle and Herbold 1987, Moyle 2002); however, given the many alterations to historical habitat conditions and species invasions (Brown and Moyle 2005, Whipple et al. 2012), it is unclear if this resistance or resilience has been maintained. We hypothesize that native species in the system currently have low drought resistance due to habitat loss and associated loss of refugia; however, we expect these species to continue to exhibit high drought resilience. In contrast, we hypothesize that nonnative species, which often thrive in highly disturbed ecosystems, would have high drought resistance and little response in post‐drought periods (Marchetti and Moyle 2001, Bêche et al. 2009).

## Methods

### Study area

**Fig. 1:**
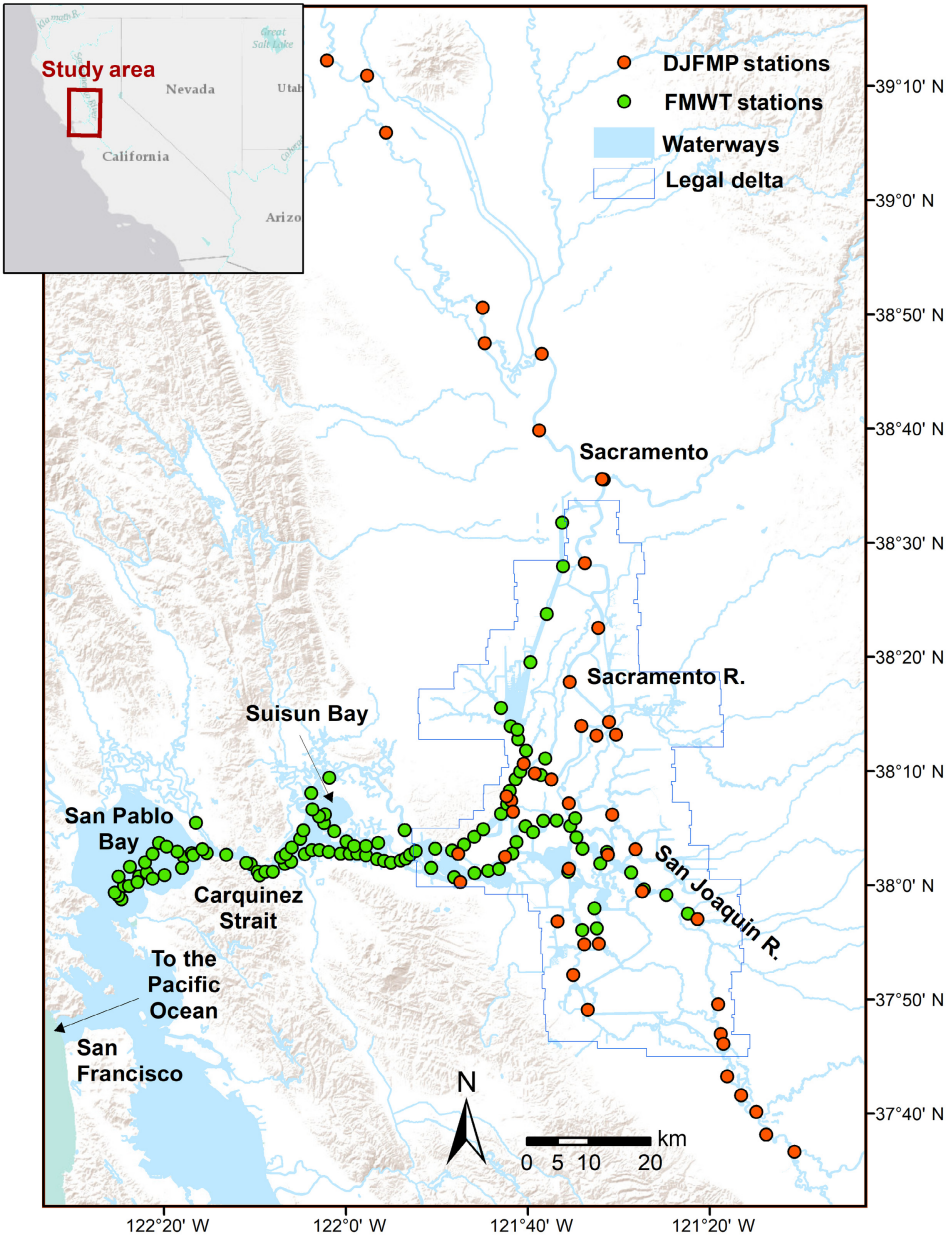
Map of the San Francisco Estuary and the Sacramento‐San Joaquin Delta, California, USA, indicating all sampling locations for the California Department of Fish and Wildlife's Fall Midwater Trawl (FMWT) survey and United States Fish and Wildlife Service's Delta Juvenile Fish Monitoring Program (DJFMP) beach seine survey used in this study.

The SFE is the largest estuary on the Pacific Coast of the United States, stretching from the tidal saline San Francisco Bay to the tidal freshwater Sacramento‐San Joaquin Delta (Delta; Fig. 1). Freshwater flows enter the SFE from the Sacramento and San Joaquin Rivers and smaller tributaries and pass through the Delta, a network of leveed channels and tidal lakes, before draining into the San Francisco Bay. The Sacramento and San Joaquin river system watershed encompasses 163,000 km^2^ and is bound by the Sierra Nevada and Cascade Ranges (Knowles 2002). The Delta was formerly a mosaic of river channels, tidal wetlands, floodplains, and riparian forests, but now consists largely of islands reclaimed for agriculture that are separated by a network of leveed channels (Nichols et al. 1986). Downstream from the confluence of these rivers, fresh water exits the Delta and enters the Suisun Bay before flowing through the narrow Carquinez Strait into San Pablo Bay, passing under the Golden Gate Bridge and into the Pacific Ocean. The SFE has socioeconomic and ecological importance by providing drinking water to 25 million people, irrigation water to a $36 billion per year agricultural industry, habitat for threatened and endangered species, and critical wintering habitat for millions of birds on the Pacific Flyway (Service 2007, Cloern et al. 2011).

The SFE has a Mediterranean climate with wet winters and warm, dry summers. Precipitation is highly variable and occurs over relatively few days in a given year (Dettinger and Cayan 2014, Dettinger et al. 2016). It is one of the most modified estuaries in the world (Nichols et al. 1986, Cohen and Carlton 1998) with highly managed freshwater input from upstream reservoirs and highly managed outflows to the ocean. Outflows are affected by the Central Valley Project and State Water Project pumping plants, which divert water from the Delta to Central and Southern California for municipal and agricultural use (Nichols et al. 1986). Additionally, there are thousands of smaller water diversions in the SFE that meet local water demands. Thus, water management strongly influences the volume of outflow from the Delta, which in turn, dictates the distribution of the salinity field (Knowles 2002). Reservoir releases and freshwater export operations vary interannually and seasonally and tend to covary.

### Data

**Fig. 2:**
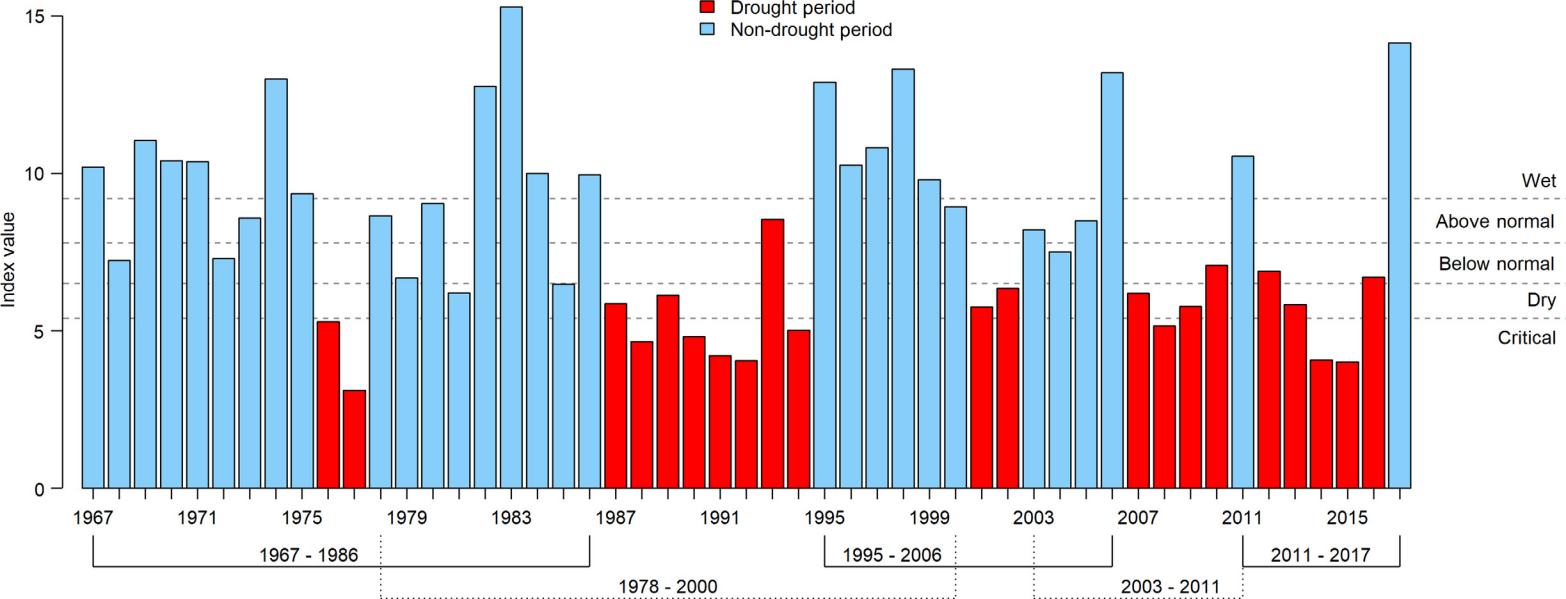
Time series of California's Sacramento Valley water year index with classifications defined by the California Department of Water Resources. Colors distinguish drought and wet periods, and bottom brackets show the five drought cycles, each of which consists of a pre‐drought wet period, a drought period, and a post‐drought wet period.

We identified drought periods using California Department of Water Resources (CDWR) water year hydrologic classification index (*available online*).^8^ Due to California's Mediterranean climate, water year is used to describe the region's interannual variability in precipitation. California's water year begins 1 October of the previous calendar year, when the wet season starts, and ends 30 September of the named water year, when the dry season ends (e.g., water year 2017 begins in October of 2016 and ends in September of 2017). The Sacramento Valley water year index was specifically used for this study because the Sacramento River provides a substantial majority of freshwater inflow into the Sacramento‐San Joaquin Delta and the SFE (Lund 2016). The Sacramento Valley water year index is a composite index based on the sum of unimpaired flows from various streams within the Sacramento River watershed and conditions from the previous year (as previous conditions affect how much water is released from reservoirs into the estuary). Using this index, we grouped all years from 1967 to 2017 into drought and wet periods (Fig. 2). We defined drought as a period that consisted of two or more consecutive water years classified as below normal, dry, or critically dry. Any non‐drought years were considered wet periods consisting of one or more years. The only exception was for the above normal water year 1993, which was included as part of the 1987–1994 drought period due to the two critically dry years (1992 and 1994) surrounding it (Fig. 2). Although we acknowledge that drought exists in a continuum (see Table 1), the classification of years into drought and non‐drought periods was necessary for our resistance and resilience modeling framework.

**Table 1:** Summary table of the hydrologic variables we evaluated to compare drought and wet years as seen in Fig. 2.

Category and variable	Description
Magnitude	
Water Year Index	Annual hydrologic index for the Sacramento Valley of California. Combination of annual runoff of current water year and previous water year. Unit is million acre‐feet (1 acre‐foot = 1,233.48 m^3^)
Total annual delta precipitation	Approximate total precipitation within the Sacramento‐San Joaquin Delta for the year. Unit is cubic feet per second (cfs; 1 cubic foot = 0.028 m^3^)
Delta inflow	Surface water inflow into the Sacramento‐San Joaquin Delta. Unit is cfs
Maximum daily inflow	Maximum daily flow into the Sacramento‐San Joaquin Delta for the water year. Unit is cfs
Duration	
Number of days with inflow over 10,000 cfs	Number of days within a water year with Delta inflow above 10,000 cfs
Timing	
Centroid day of outflow	Outflow is Delta inflow adjusted for water export (see mean daily export). Timing of peak outflow was estimated by calculating the centroid of outflow distribution based on number of days since 1 October similar to center of distribution calculation in Dege and Brown (2004). Unit is the number of days since 1 October
Centroid day of precipitation	Timing of peak precipitation within a water year, see above. Unit is the number of days since 1 October
Anthropogenic changes	
Mean daily exports	Estimated mean daily water export from the Sacramento‐San Joaquin Delta. Unit is cfs
Spatial variability	
SJR : SAC ratio	Total flow from the San Joaquin River divided by total flow from the Sacramento River for the water year. Sacramento and San Joaquin Rivers are the two primary tributaries that drain into the San Francisco Estuary
Temporal variability	
Standard deviation of inflow	Standard deviation calculation of daily inflow into the Sacramento‐San Joaquin Delta. Unit is cfs

We used occurrence data from the two long‐term fish monitoring programs to assess the relative resistance and resilience to drought for key fish species in the SFE. Pelagic fish data were obtained from the California Department of Fish and Wildlife's Fall Midwater Trawl (FMWT) monitoring program and littoral fish data were obtained from the United States Fish and Wildlife Service's Delta Juvenile Fish Monitoring Program (DJFMP). The FMWT has conducted monitoring for juvenile fishes in the open water (i.e., pelagic) habitat of the SFE since 1967 (Stevens and Miller 1983). The original goal of FMWT was to monitor the annual relative abundance of young‐of‐year striped bass (*Morone saxatilis*); however, over the years it has provided valuable information on the endangered delta smelt (*Hypomesus transpacificus*) and other species of management interest (Moyle et al. 1992, Feyrer et al. 2007, 2009, Rosenfield and Baxter 2007, Bever et al. 2016, Nobriga and Rosenfield 2016). The FWMT has sampled 100 stations from San Pablo Bay landward into the Sacramento‐San Joaquin Delta once per month from September to December since 1967 (Fig. 1) and it has sampled an additional 22 stations per month since 2010 (Appendix S1: Fig. S1, Table S1).

The DJFMP has conducted beach seine surveys since 1976 to evaluate the abundance and distribution of juvenile Chinook salmon (*Oncorhynchus tshawytscha*) and various resident fish species within the SFE (IEP et al. 2019). The DJFMP beach seine survey has been the primary monitoring program in the region that evaluates fish community changes in the nearshore, littoral habitat (Brown and May 2006, Mahardja et al. 2017a). Although DJFMP began in 1976, sampling in the late spring and summer months (when non‐salmonid juvenile fishes typically recruit into the gear) did not become part of standard protocol until 1995. Since 1995, DJFMP has sampled 44 sites within the Sacramento‐San Joaquin Delta and the lower Central Valley of California in a consistent manner year‐round (Fig. 1; Appendix S1: Fig. S2, Table S2). Beach seine sampling at each site was conducted either weekly or biweekly depending on the region and logistical constraints (IEP et al. 2019). For non‐salmonid species within the DJFMP data set, we limited the data to the years 1995 and after, and months from March to August similar to Mahardja et al. (2017) because these are the years and months that had consistent sampling for most non‐salmonid juvenile fishes.

We selected fish species based on their importance to the management of the estuary and how commonly the fish species was caught in the monitoring programs (Stevens and Miller 1983, Sommer et al. 1997, 2007, Brown and May 2006, Mahardja et al. 2016, 2017a). Data for six fish species were used from the FMWT data set: striped bass, delta smelt, longfin smelt (*Spirinchus thaleichthys*), threadfin shad (*Dorosoma petenense*), and American shad (*Alosa sapidissima*; Stevens and Miller 1983, Sommer et al. 2007). For the DJFMP beach seine survey data set, we used data for seven fish captured fairly regularly by the monitoring program: Sacramento splittail (*Pogonichthys macrolepidotus*), Mississippi silverside (*Menidia audens*), largemouth bass (*Micropterus salmoides*), Sacramento sucker (*Catostomus occidentalis*), bluegill (*Lepomis macrochirus*), redear sunfish (*Lepomis microlophus*), and Chinook salmon (Sommer et al. 1997, Brown and May 2006, Mahardja et al. 2016, 2017a). Because consistent sampling for the DJFMP beach seine survey did not begin until 1995, we were only able to assess occurrence changes of the three littoral species for the three most recent drought cycles (Fig. 2). We inspected the fork length distribution of each species, and removed catch of larger‐sized fish prior to analysis to ensure that our results pertain to mostly young‐of‐years (Table 2). We used data from every station that contained at least a single sampling occasion in each of the three major periods (the initial wet period, drought, and the recovery period) for at least one of the drought cycles (Appendix S1: Table S2).

**Table 2:** List of fish species analyzed in this study along with their life history characteristics (Moyle 2002, Nobriga et al. 2005) and size cut‐offs used for our analysis.

**Species**	Origin	General maximum size (fork length in mm)	Average age at maturation (yr)	Maximum age (yr)	Maximum fecundity	Migratory pattern	Habitat	Fork length cut‐off (mm)
American shad (*Alosa sapidissima*)	Nonnative	600	4	7	225,600	Anadromous	Pelagic	150
Delta smelt (*Hypomesus transpacificus*)	Native	120	1	2	12,000	Semi‐anadromous	Pelagic	150
Longfin smelt (*Spirinchus thaleichthys*)	Native	150	2	3	24,000	Anadromous	Pelagic	150
Striped bass (*Morone saxatilis*)	Nonnative	1,250	4	30	5,000,000	Anadromous	Pelagic	NA†
Threadfin shad (*Dorosoma petenense*)	Nonnative	220	1	3	21,000	Resident	Pelagic	150
Splittail (*Pogonichthys macrolepidotus*)	Native	450	2	8	100,000	Semi‐anadromous	Littoral	100
Mississippi silverside (*Menidia audens*)	Nonnative	120	1	2	15,000	Resident	Littoral	150
Largemouth bass (*Micropterus salmoides*)	Nonnative	760	2	16	94,000	Resident	Littoral	175
Sacramento sucker (*Catostomus occidentalis*)	Native	560	5	30	32,300	Resident	Littoral	100
Bluegill (*Lepomis macrochirus*)	Nonnative	260	2	6	50,000	Resident	Littoral	200
Redear sunfish (*Lepomis microlophus*)	Nonnative	254	2	7	80,000	Resident	Littoral	200
Chinook salmon (*Oncorhynchus tshawytscha*)	Native	1,000	3	5	17,000	Anadromous	Littoral (at fry stage)	55

† NA, not applicable. Striped bass data had already been filtered to just young‐of‐year fish by the FMWT survey.

Additional considerations must be made when using juvenile Chinook salmon data in the DJFMP beach seine survey data set. Hatcheries continue to play a major role in the management of Chinook salmon within the SFE system (Huber and Carlson 2015, Willmes et al. 2018, Sturrock et al. 2019). However, since 1999, hatcheries in California's Central Valley discontinued the release of smaller‐sized Chinook salmon into the system (those below 55 mm fork length). In order to focus on the ecological response of naturally produced Chinook salmon and reduce the influence of hatchery fish within the data set, we used only smaller‐sized Chinook salmon data within the fry life stage (<55 mm fork length) for years 1999 and after, per Munsch et al. (2020). We acknowledge that Chinook salmon exhibit a diverse life history strategy and that we would miss smolt outmigrants by filtering our data in such a way (Sturrock et al. 2020). However, it is advantageous in that Chinook salmon fry are more commonly found in nearshore habitat sampled by beach seine (Munsch et al. 2016). For Chinook salmon, we used data from December to May, the period in which we would see juveniles in the study area. We follow the convention of California's water year to describe years for Chinook salmon (e.g., December 1998 to May 1999 will simply be referred to as the year 1999).

### Data analysis

To ensure the validity of our drought and wet period classifications and better understand how they differ, we conducted an ANOVA to test for significant differences between wet and dry periods using hydrologic variables (α = 0.05; Table 1). This was done by using the aov function for each hydrological variable in the R programming language (R Core Team 2018). The hydrologic variables represented the timing, duration, magnitude, and variability of flow events, and were obtained from the DAYFLOW database (*available online*).^9^

We defined species' resistance to drought as the lack of a large‐scale decline in occurrence from a wet period to a drought period. Species' resilience to drought was defined as the return of occurrence to wet period levels following a drought period. To assess changes in occurrence from the initial wet period to the drought period and from the drought period to subsequent recovery wet period (Fig. 2), we used a Bayesian logistic regression model with a framework similar to that found in Bennett et al. (2014). We extended the model of Bennett et al. (2014) to include multiple drought cycles.

Let *c* = 1, …, 5 represent drought cycle number and let *d* = 1, 2, 3 represent the period within a drought cycle with 1 = pre‐drought wet period, 2 = drought period, and 3 = post‐drought wet period. We constructed a separate model for each species with the following structure:

(1) $$y_{c,d,r,m,s,i}∼\mathrm{Binomial}\left(1,p_{c,d,r,m,s}\right)$$

where $y_{c,d,r,m,s,i}$ represents the presence (*y* = 1) or absence (*y* = 0) of the species in sample *i* at station *s* in month *m* and year *r* within period *d* of drought cycle *c* and $p_{c,d,r,m,s}$ is the rate of occurrence. For the first drought cycle this rate is modeled as

(2) $$\mathrm{logit}\left(p_{c=1,d,r,m,s}\right)=\alpha +\alpha_{r}+\alpha_{m}+\alpha_{s}+\Delta_{1,c=1}I_{d>1}+\Delta_{2,c=1}I_{d>2}$$

where α is an overall mean occurrence rate (on the logit scale) for the first period; α*_r_*, α*_m_*, and α*_s_* are random intercepts corresponding to year, month, and station; and $\Delta_{1,c=1}$ and $\Delta_{2,c=1}$ are the resistance and resilience coefficients for the first drought cycle. The binary indicator variable $I_{d>1}$ takes the value 1 in the second (drought) period and after in the time series and 0 otherwise; similarly, $I_{d>2}$ takes the value 1 in the third (post‐drought) period and after in the time series and 0 otherwise. Note that the first four terms on the right side of Eq. 1 define the logit‐scale rate of occurrence in the first period, $\mathrm{logit}\left(p_{c=1,d=1,r,m,s}\right)=\alpha +\alpha_{r}+\alpha_{m}+\alpha_{s}$, which serves as the reference level for periods two and three.

For drought cycles two through five, a given cycle's pre‐drought wet period (*m* = 1) is identical to the previous cycle's post‐drought wet period (*m* = 3; Fig. 2). Using the latter as a reference level for the former, the occurrence rate for *c* = 2, …, 5 can then be written as

(3) $$\mathrm{logit}\left(p_{c,d,r,m,s}\right)=\mathrm{logit}\left(p_{c‐1,d=3,r,m,s}\right)+\Delta_{1,c}I_{d>1}+\Delta_{2,c}I_{d>2}.$$

Prior distributions for the model parameters were

$$\alpha_{r}∼N\left(0,\sigma_{r}\right)$$

$$\alpha_{m}∼N\left(0,\sigma_{m}\right)$$

$$\alpha_{s}∼N\left(0,\sigma_{s}\right)$$

$$\sigma_{r}∼\mathrm{HalfCauchy}\left(0,1\right)$$

$$\sigma_{m}∼\mathrm{HalfCauchy}\left(0,1\right)$$

$$\sigma_{s}∼\mathrm{HalfCauchy}\left(0,1\right)$$

$$\alpha ∼N\left(0,10\right)$$

$$\Delta_{1,c}∼N\left(0,10\right),\;\text{for}\;c=1,\ldots ,5$$

$$\Delta_{2,c}∼N\left(0,10\right),\;\text{for}\;c=1,\ldots ,5.$$

The overall intercept, α, as well as the resistance and resiliency coefficients, were given weakly informative normal priors. All random intercept parameters were also assigned weakly informative normal priors with mean 0 and standard deviation given by a half‐Cauchy distribution (Polson and Scott 2012). We considered there to be strong evidence for lack of drought resistance if the upper limit of the 95% credible interval for the resistance coefficient was below zero. Similarly, strong evidence for resilience in the subsequent wet period was defined by the lower limit of the 95% credible interval for the resilience coefficient being above zero. We ran all models using Hamiltonian Monte Carlo in R (R Core Team 2018) and Stan (Carpenter et al. 2017, Stan Development Team 2020) with the rethinking package (McElreath 2018; Data S1). Models were estimated with four independent chains of 15,000 iterations each after 5,000‐iteration burn‐in periods. Adequate mixing and convergence were evaluated by inspecting chain histories and verifying that potential scale reduction factors were near one. Model goodness‐of‐fit was evaluated by comparing 2,000 samples from the marginal posterior distribution for *y* with the actual observations and summarizing the mean accuracy rates across observations.

## Results

From 1967 to 2017, we identified five drought periods: 1976–1977, 1987–1994, 2001–2002, 2007–2010, and 2012–2016 (Fig. 2). We found significant differences (*P* < 0.01) between wet and drought periods for the majority of the hydrologic variables tested (Table 3). Wet years had more days with high flow, more precipitation, elevated flows of water into the Delta (both on a daily and an annual basis), more even flow ratio between the two major tributaries of Sacramento and San Joaquin Rivers, and flow intra‐annual variability. We did not find strong differences in timing of peak precipitation and outflow between drought and non‐drought years (Table 3; Appendix S1: Figs. S3, S4). Similarly, total volume of anthropogenic water exports away from the Delta did not differ between drought and non‐drought years (Table 3; Appendix S1: Fig. S5).

**Table 3:** ANOVA results for hydrologic variables used to distinguish drought vs. non‐drought years as denoted in Fig. 2.

Category and variable	ANOVA results				Mean values	
	*F*	df	*P*	MSE	Non‐drought	Drought
Timing						
Centroid day of outflow	1.14	(1, 48)	0.29	296	157	162
Centroid day of precipitation	1.90	(1, 48)	0.17	241	119	125
Duration						
Number of days with inflow over 10,000 cfs	73.4	(1, 48)	<0.01	3,720	246	96
Magnitude						
Water Year Index	62.5	(1, 48)	<0.01	3.74	9.9	5.5
Total annual precipitation	22.7	(1, 48)	<0.01	18	524,286	341,117
Inflow	45.0	(1, 48)	<0.01	1.92 × 10^8^	43,872	17,220
Maximum daily inflow	26.6	(1, 48)	<0.01	1.40 × 10^10^	238,288	63,412
Location						
SJR : SAC ratio	18.6	(1, 48)	<0.01	6.45 × 10^−3^	0.21	0.11
Anthropogenic changes						
Mean daily exports	0.49	(1, 48)	0.49	3.41 × 10^6^	6,370	5,998
Variability						
Standard deviation of inflow	36.4	(1, 48)	<0.01	3.21 × 10^8^	39,808	8,848

**Fig. 3:**
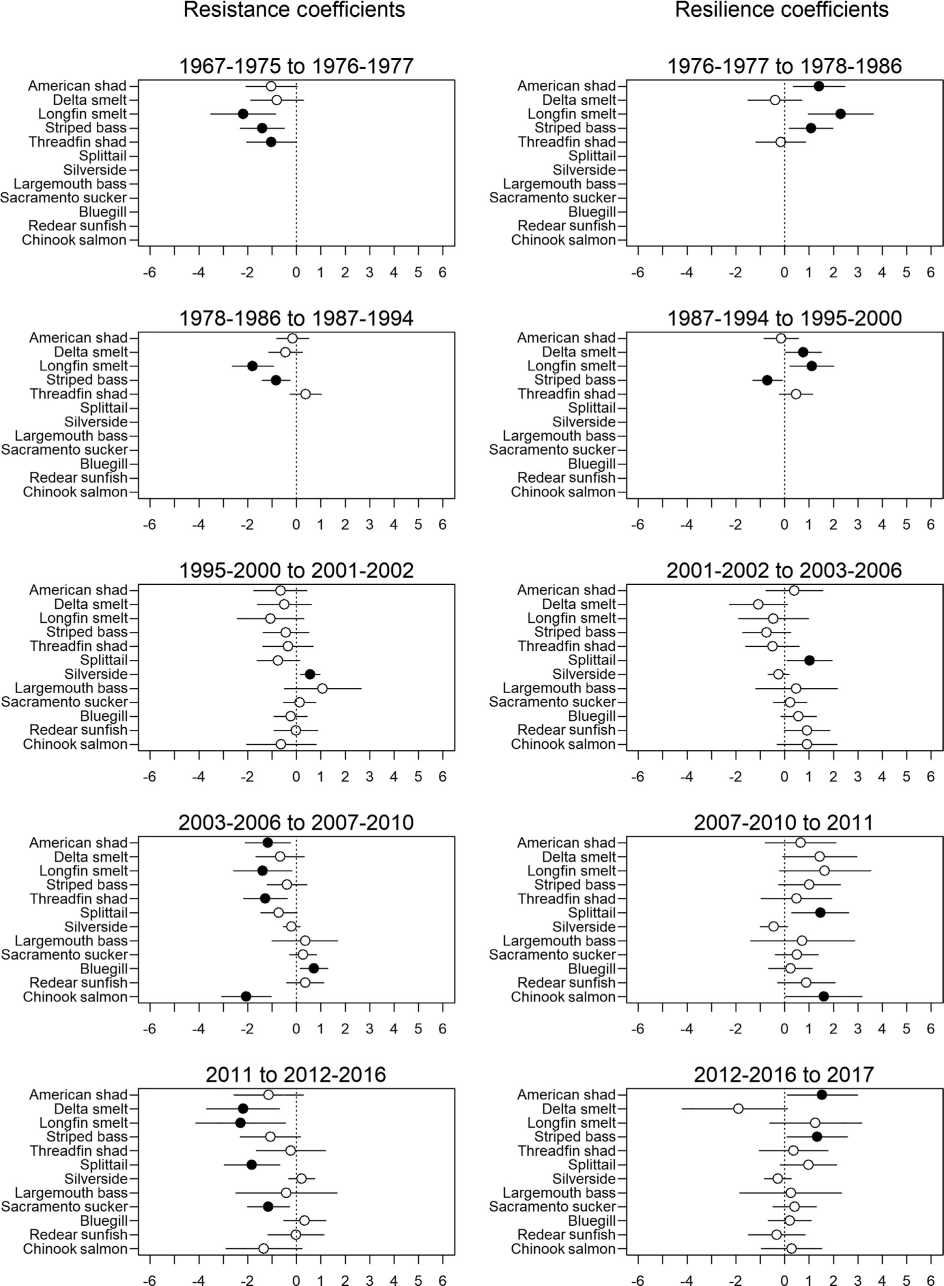
Resistance and resilience coefficients for each species and drought cycle. Left column shows the drought resistance coefficient while the right column shows the drought resilience coefficient. Positive resistance coefficient indicates increase in occurrence from pre‐drought to drought period. Positive resilience coefficient indicates increase in occurrence from drought period to the post‐drought (i.e., recovery) period. Lines extending from each point indicate the 95% credible intervals for each term. Black points indicate coefficients with 95% credible intervals that do not include zero.

**Fig. 4:**
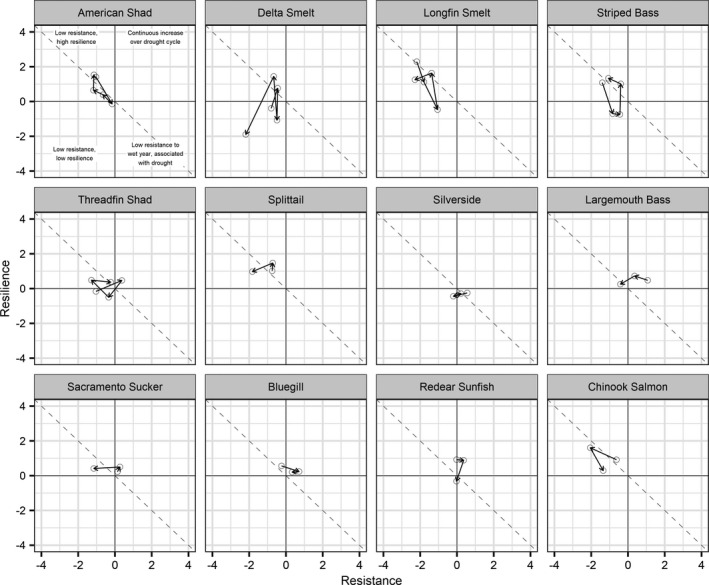
Summary of mean drought resistance and resilience coefficients for all drought cycles, sorted by species. Each circle represents the estimated mean resistance and resiliency coefficients for a drought cycle. The direction of arrow points to the next drought cycle in the time series. Dashed diagonal line indicates the complete post‐drought recovery of species if low resistance to drought was observed (i.e., decline during drought period).

Accuracy rates from the nine logistic regression models were fairly high with means ranging from 0.61 to 0.87, and medians ranging from 0.64 to 0.96 (Appendix S1: Table S3). There was strong evidence of decline in fish occurrence (upper 95% credible interval for resistance coefficient under 0) in 28% of all drought period and species combinations (Fig. 3). Meanwhile, apparent decline (resistance coefficient < 0) during the drought made up 78% of all cases. The occurrence of pelagic species such as delta smelt, longfin smelt, striped bass, and American shad never increased during a drought in the 50‐yr period covered in our study (1967–2017). We found only 10 instances in which we observed no apparent decline during a drought period (resistance coefficient > 0) for all fish. All but one of these cases involved nonnative species, and similarly, all but one involved littoral species (Fig. 4; Appendix S1: Fig. S6). Sacramento sucker was the only native species with any positive mean resistance coefficient, while threadfin shad was the only pelagic species with any positive mean resistance coefficient. There was only a single observation of positive mean resistance coefficient for threadfin shad out of the five drought cycles, which occurred during the second drought cycle (1978–2000). We observed a substantial increase in occurrence during the post‐drought, recovery wet period (lower 95% credible interval for resilience coefficient over 0) in 22% of all cases. Apparent increase in the recovery wet period was observed in 72% of cases.

We found 13 out of 46 cases had a strong evidence of decline in species occurrence during a drought. Strong evidence of recovery was observed in 4 out of these 13 cases (31%; Fig. 3). Under this low resistance and high resilience group, full recovery (defined as mean resilience coefficient estimate being equal to or higher than resistance coefficient estimate) occurred in two out of four cases. The two cases were for longfin smelt, one in the first drought cycle of 1967–1986 and another in the fourth drought cycle of 2003–2011 (Fig. 4; Appendix S1: Fig. S6). We found no strong evidence of species increase during both the drought and recovery wet period based on our 95% credible interval criteria; however, we did observe eight instances both positive resistance and resilience coefficients within a drought cycle: threadfin shad during the second drought cycle (1978–2000), largemouth bass, bluegill, and Sacramento sucker during the third drought cycle (1995–2006), and largemouth bass, bluegill, redear sunfish, and Sacramento sucker during the fourth drought cycle (2003–2011). In a couple of instances, we observed an inverse relationship to drought (increase during drought, decrease during subsequent wet period) with Mississippi silversides. We did not see clear patterns between drought resistance, resilience, and any particular life history characteristics (Table 2); however, of the species we analyzed, we only observed positive mean resistance coefficients for resident fish species.

## Discussion

Climate change will inevitably impact estuaries in significant ways; however, there are many uncertainties given the highly dynamic and complex nature of estuaries and the need to integrate the effects of changes in both oceanic and inland ecosystems. In this study, we sought to better understand how estuarine fish species respond to extreme events such as droughts, which are predicted to become more common due to climate change. Despite the continuous changes that have occurred in the SFE system of California, our results indicate that there exists unifying themes connecting the multiple drought periods that have occurred over the past 50 yr. These patterns are most obvious when considering the origin (native vs. nonnative) and habitat (pelagic vs. littoral) of fishes that we selected for our analysis (Table 2).

Drought periods can be characterized as having less flow, as well as shorter duration and lower magnitude of peak flows relative to recovery periods. During droughts, there is also a shift in the relative contributions of the major water sources for the estuary, which could contribute to changes in water quality. The weight of evidence from our models suggests that the occurrence of young‐of‐year and annual pelagic fishes in the SFE consistently decline during droughts, regardless of whether they are native or nonnative. Pelagic fish species also demonstrated resilience in most cases, where occurrence levels returned to pre‐drought values. Yet full recoveries do not occur in every drought cycle, leading to permanently lower baseline numbers over the –50‐yr study period, a finding consistent with earlier studies (Mac Nally et al. 2010, Thomson et al. 2010). In contrast, a considerable portion of littoral fish species in SFE, most of which are nonnative, were not only more resistant to drought, but their occurrence may increase during certain drought periods. Unlike studies that have demonstrated persistence of nonnative fishes during droughts (Bêche et al. 2009, Bezerra et al. 2018, Ramírez et al. 2018, Rogosch et al. 2019), our results indicate that drought responses in SFE are more habitat‐specific and not necessarily driven just by native and nonnative species differences.

There are several factors to consider when interpreting our findings. By design, our study did not evaluate the severity of each drought period. The capacity of species to recover is likely a function of the duration of the drought. Nevertheless, we observed some similarities across the five drought cycles despite variability in drought severity. We also acknowledge that the data underlying our analysis did not always cover the full spatial extent of every species included in the study. For example, longfin smelt can be present downstream of the sampling area of our study (Rosenfield and Baxter 2007, Lewis et al. 2020). A small, distinct population of Sacramento splittail commonly found in the Petaluma and Napa Rivers is also likely not well‐represented in the data we used for this study (Baerwald et al. 2007, Mahardja et al. 2015). In addition, spawning for some species (e.g., Sacramento sucker, striped bass, American shad) occur in upstream tributaries not sampled in our study area and a portion of their young‐of‐year may continue to rear in these upstream areas (Moyle 2002). The imperfect spatial coverage of our data may bias our resistance and resiliency estimates to some extent; however, most of the juvenile fishes we analyzed either migrate through or occur largely within our study area, and the patterns described in our study are consistent with large‐scale changes in the populations of these fish species documented by other monitoring programs (Brown and Michniuk 2007, Feyrer et al. 2015, Polansky et al. 2019). Last, our analysis did not account for catchability or detection probability, which can often vary considerably over time and space (MacKenzie et al. 2002). Some studies have suggested that the overall decline of turbidity in the SFE (Hestir et al. 2016) have caused some amount of reduction in the catch probability of fishes through gear avoidance (Latour 2016, Peterson and Barajas 2018). However, it is impossible to differentiate the relative contribution of turbidity on abundance vs. catch probability based on field data alone. It seems unlikely that the level of population declines and recoveries observed in the SFE would be predominantly driven by gear avoidance (Tobias 2020). The overall patterns seen in this study are generally consistent with those observed in larval fish surveys where swimming capabilities of fishes would be of less concern (Dege and Brown 2004), other sampling methods that we expect to be unaffected by turbidity (Grimaldo et al. 2009), and with studies that have adjusted for fish catchability (Mahardja et al. 2017b, Peterson and Barajas 2018, Polansky et al. 2019).

Understanding species' vulnerability to drought and their capability to recover is key information for the conservation of native fish species within the SFE. With the exception of Sacramento sucker, the native fish species we analyzed seemed to have low resistance to droughts but demonstrate resilience in most drought cycles (Table 2, Fig. 3). Resilience in the native fishes of SFE appears to be contingent on the suite of environmental factors critical to each species and how they relate to the increased flow during post‐drought periods. The SFE‐endemic Sacramento splittail demonstrated low resistance to drought, but consistently recovered during subsequent wet years (Fig. 4). This is consistent with the current understanding that the relatively long‐lived Sacramento splittail (Daniels and Moyle 1983) depend on strong year classes that are recruited during wet years when floodplain habitat is available for spawning (Sommer et al. 1997, Moyle et al. 2004). Similarly, Chinook salmon exhibited low resistance and high resilience, likely due to increased survival of juveniles in freshwater in wet years (Michel et al. 2015) and availability of high‐quality floodplain habitat (Sommer et al. 2001, Goertler et al. 2018). The low resistance and relatively high resilience of the native Longfin Smelt is also expected given the positive influence of freshwater flow on juvenile production (Rosenfield and Baxter 2007, Nobriga and Rosenfield 2016). The flow‐related mechanism that modulates longfin smelt abundance is not particularly well understood, but it is hypothesized that low‐to‐moderate salinity and high turbidity throughout large parts of the SFE during wet years create suitable habitat for the species (Grimaldo et al. 2017, Mahardja et al. 2017b). Similar to longfin smelt, the distribution of the threatened delta smelt has been linked to freshwater flow based on the expansion of low salinity habitat during wet years (Feyrer et al. 2007, 2011, Kimmerer et al. 2013, Bever et al. 2016). However, the inability of delta smelt to rebound in only two out of the last five post‐drought periods may be due to water quality. High temperature has been demonstrated to be a limiting factor for delta smelt in bioassays (Swanson et al. 2000, Davis et al. 2019), and above‐average summer temperatures in SFE may have contributed to their lack of post‐drought recovery in recent wet years (Brown et al. 2013, 2014, 2016). Other unmeasured habitat attributes likely also play a role in the lack of delta smelt recovery, but they are beyond the scope of our study. Unlike other native fishes of the SFE, the Sacramento sucker exhibited relatively high drought resistance (Figs. 3, 4). The high resistance of Sacramento sucker is likely due to its extended life span (Table 2). Stream populations of this species can be dominated by large adults from years that were favorable for juvenile survival (Moyle et al. 1983). These dominant year classes can persist for long periods. The adults spawn every year producing young‐of‐year that provide evidence of persistence in our study, but recruitment is episodic, depending on environmental conditions. Higher survival of young‐of‐year in wet years after a stressful drought results in higher measures of resilience. This life history is common among large, long‐lived cyprinids and catostomids (Moyle and Herbold 1987).

The impact of drought on nonnative fish occurrence varied based on habitat. Nonnative pelagic fishes of the SFE (threadfin shad, American shad, and striped bass) generally exhibited low drought resistance and high resilience during our study period. However, these nonnative pelagic fish species did not demonstrate synchronous decline and rebound throughout every drought cycle (Appendix S1: Fig. S6). There is a general paucity of information on the flow‐related mechanisms that would affect the abundance and distribution of these species; however, previous studies indicated that availability of suitable freshwater habitat may increase their occurrence during wet years (Feyrer et al. 2007, Kimmerer et al. 2009, 2013). The nonnative littoral fish species included in our analysis (largemouth bass, bluegill, redear sunfish, and Mississippi silverside) are generally considered warm‐water and drought‐tolerant species, and, as such, it is not surprising that they rarely showed decline during droughts (Rypel 2009, Davis et al. 2019). Nevertheless, there is a notable difference in how these species respond to drought. Numbers of largemouth bass, bluegill, and redear sunfish seem to have progressively increased between 1995 and 2011 (Fig. 4; Mahardja et al. 2017a), possibly due to the expansion of invasive submerged aquatic vegetation in the upper SFE over the past decade or two that have been associated with drought (Khanna et al. 2015, Conrad et al. 2016, Santos et al. 2016, Kimmerer et al. 2019). On the other hand, Mississippi silverside appears to have a negative association with freshwater flow (Mahardja et al. 2016) that led to a mostly positive drought resistance coefficient and consistently negative resiliency coefficient (Fig. 4).

In addition to interspecific variation in drought response, we observed notable differences in the general response of fishes to some drought cycles. The post‐drought recovery period of 2003–2006 was particularly striking as many species continued to decline after the end of the drought period. This post‐drought period took place after 2002, when the SFE saw an abrupt decline of multiple pelagic fish species, likely due to multiple interacting stressors such as low food availability and predation pressure (Mac Nally et al. 2010, Thomson et al. 2010). Since the 2003–2006 post‐drought period, the numbers of these SFE pelagic fish species have remained at low levels. Notably, in our time series, the failure of pelagic fish species to recover from a drought can result in a long‐term decline in occurrence. For some species, such as the threatened Delta Smelt, the lack of recovery after the most recent drought of 2012–2016 led to such low density for the species that the monitoring program used in this study failed to catch a single fish in 2018 or 2019. A new highly intensive monitoring program for Delta Smelt that was established in late 2016 demonstrated that the species is not yet extinct, but its population remained at historical low abundance level in 2019 (USFWS et al. 2019).

### Management implications

The management implications of our study are relevant to the SFE and are also instructive for other estuaries, where many similar ecological changes are occurring (Matthews and Marsh‐Matthews 2003, Cloern and Jassby 2012). Integrating our study with previous investigations on SFE ecology suggests that the increased prevalence of droughts predicted by current climate change models will shift the upper SFE fish assemblage towards more nearshore littoral fish species. Unlike many nonnative littoral fish species, the pelagic fish assemblage of the SFE appears to be largely drought‐sensitive and therefore declining. This suite of pelagic fish species includes longfin smelt, a species currently listed under the California Endangered Species Act, and delta smelt, which is listed under both the United States Endangered Species Act and California Endangered Species Act. Despite the ability of many fish species to rebound during wet years (e.g., longfin smelt, delta smelt, Chinook salmon, etc.), post‐drought recoveries sometimes did not occur. Drought did not lead to an increase in the abundance and distribution for most species during our study period, suggesting that freshwater flow remains a crucial but not necessarily sufficient component for conserving estuarine biodiversity. However, California's water resources are scarce and an ever‐growing competing demand for water lies in the future. Increased frequency of drought in the future may lead to further decline or extinction of SFE‐endemic pelagic fish species such as delta smelt and longfin smelt (Hobbs et al. 2017) and the proliferation of drought‐tolerant nonnative fish species commonly found in littoral habitat (Davis et al. 2019). It is increasingly important for water management operations to optimize water release timing and location such that they balance human and environmental needs (Chen and Olden 2017), and to do so under an adaptive management framework (Tamburello et al. 2019). Successful environmental flow management for species of concern would also require the proper consideration of other conservation measures such as habitat restoration, invasive species management, reduced contaminant loading, and climate change mitigation (Arthington et al. 2006).

## Supporting information

Appendix S1

Data S1

Metadta S1

## Data Availability

Data are available on Figshare: https://doi.org/10.6084/m9.figshare.12855581.v3

## Footnotes

^8^
https://cdec.water.ca.gov/reportapp/javareports?name=WSIHIST

^9^
https://data.cnra.ca.gov/dataset/dayflow
