# Supplementary material for: Resistance and resilience of pelagic and littoral fishes to drought in the San Francisco Estuary
Source: Ecol Appl. 2021 Jan 22;31(2):e02243. doi: 10.1002/eap.2243 (PMC7988542; doi:10.1002/eap.2243)
Supplement: Supplementary file 1 — Appendix S1 [file EAP-31-e02243-s003.pdf]

Supporting Information. Mahardja, B., V. Tobias, S. Khanna, L. Mitchell, P. Lehman, T. Sommer, L. Brown, S. Culberson, and J.L. Conrad. 2020. Resistance and resilience of pelagic and littoral fishes to drought in the San Francisco Estuary. Ecological Applications.

Appendix S1.

Additional information on data sets used for each drought cycle model and model results.

Fig. S1: Map of the San Francisco Estuary, indicating sampling locations for the FMWT survey used in this study and associated station numbers.

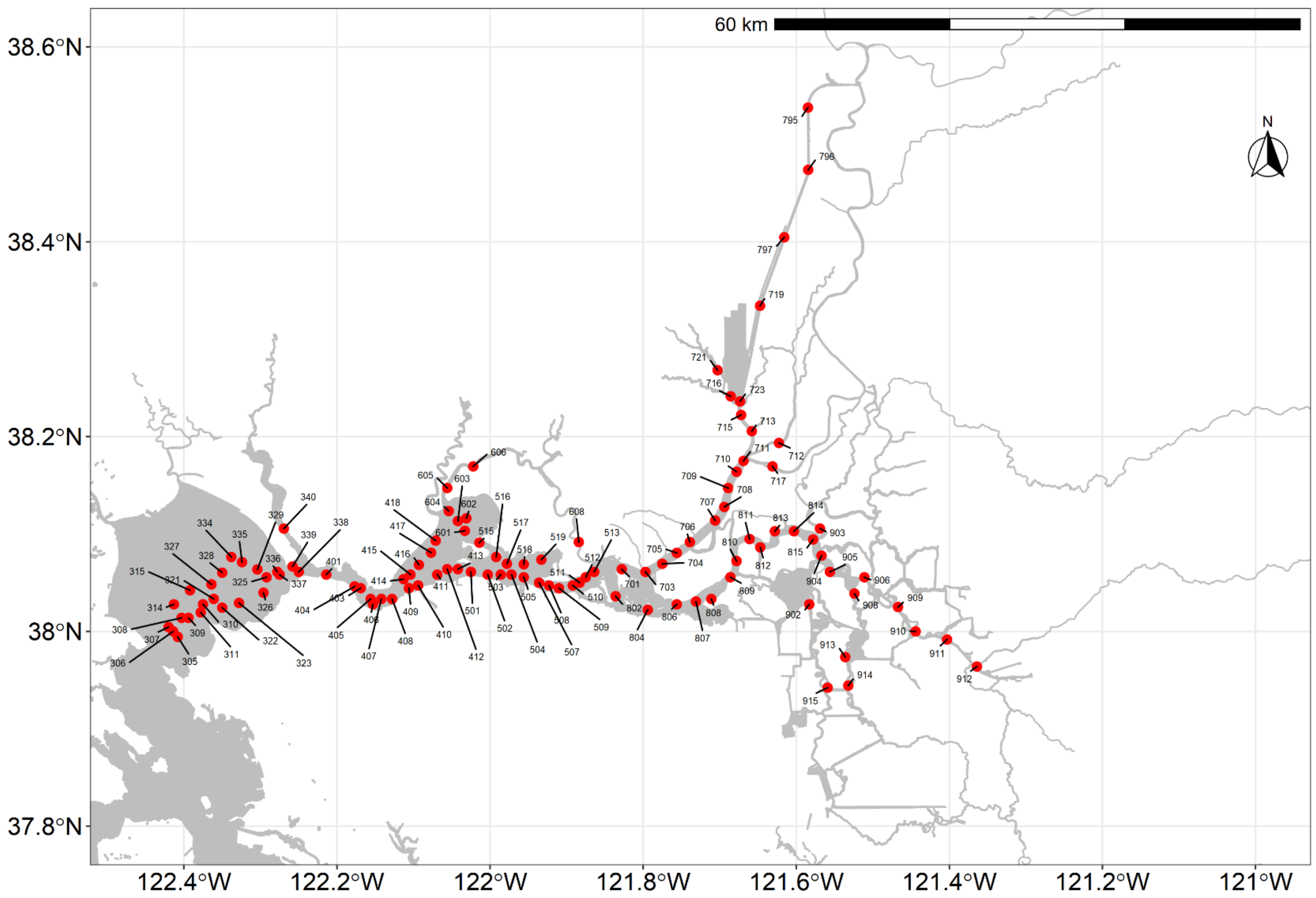

Figure S2: Map of the upper San Francisco Estuary and its watershed, indicating sampling locations for the DJFMP beach seine survey used in this study and associated station codes.

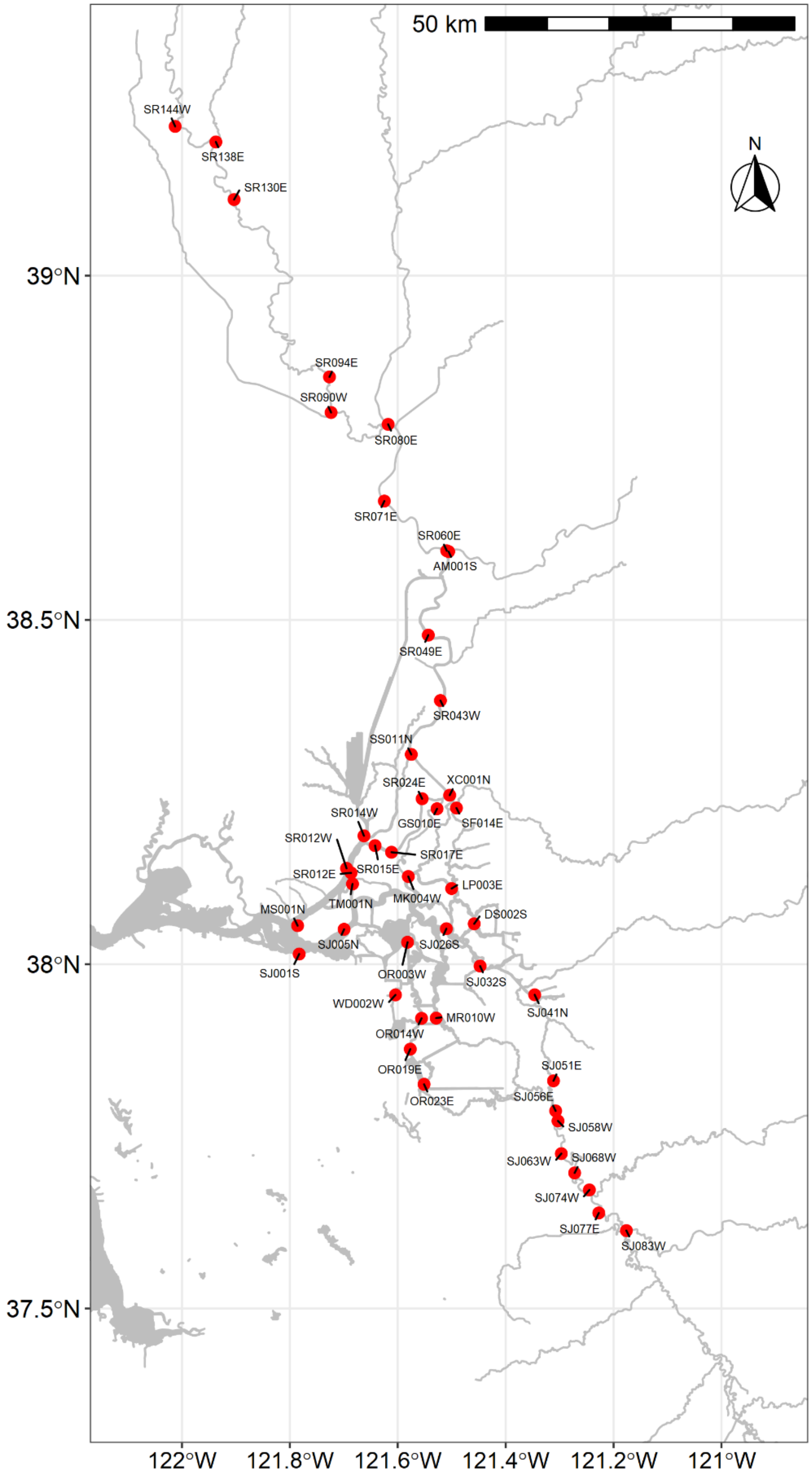

Figure S3. Annual summary of centroid day of outflow as described in Table 1 for our study period (1967-2017). Unit for y-axis is the number of days since October 1st. Water year classification was based on California Department of Water Resources index as seen in Figure 2.

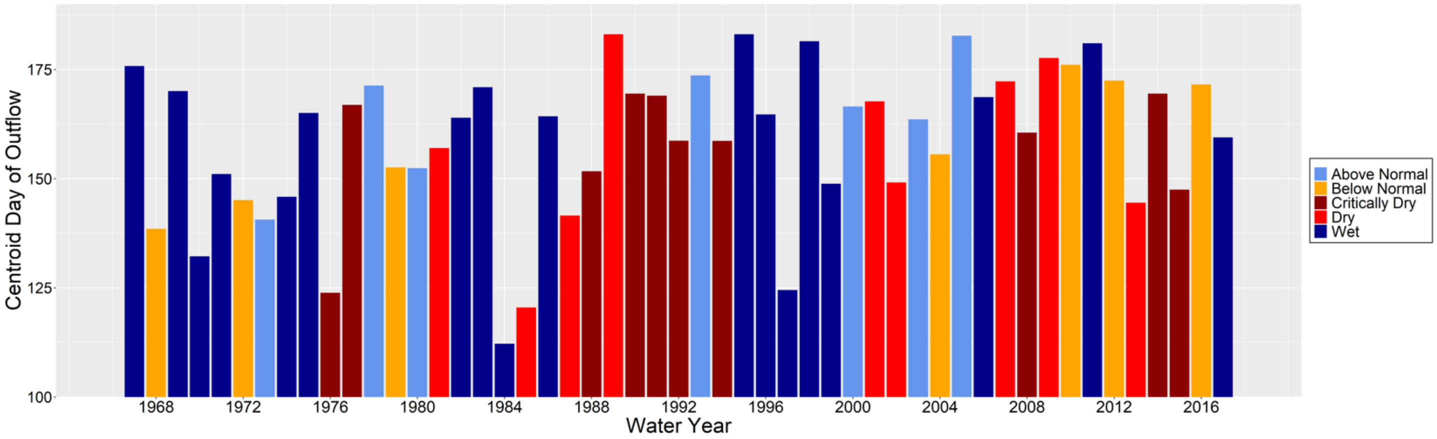

Figure S4. Annual summary of centroid day of Delta precipitation as described in Table 1 for our study period (1967-2017). Unit for y-axis is the number of days since October 1st. Water year classification was based on California Department of Water Resources index as seen in Figure 2.

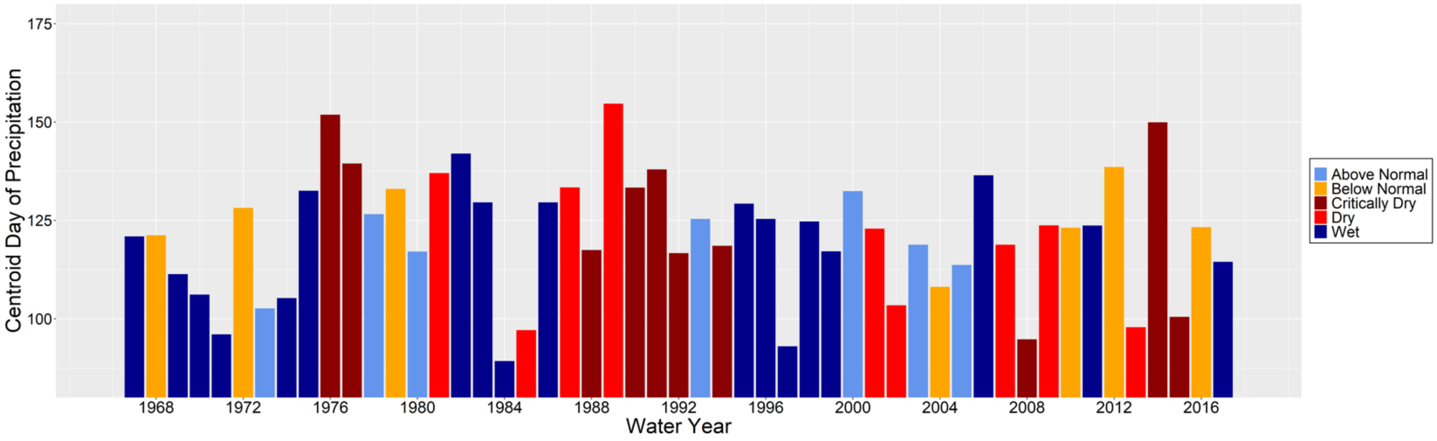

Figure S5. Annual summary of mean daily water export from the Sacramento-San Joaquin Delta as described in Table 1 for our study period (1967-2017). Unit for y-axis is in cubic feet per second. Water year classification was based on California Department of Water Resources index as seen in Figure 2.

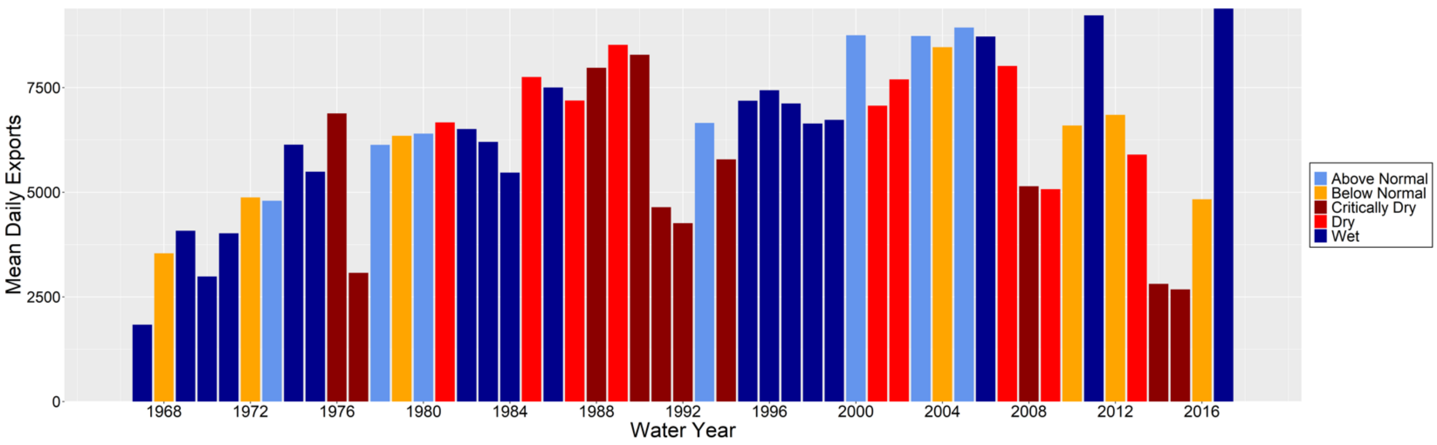

Figure S6: Resistance and resilience coefficients from Bayesian logistic regression models, sorted by species and drought cycle number. Lines extending from each point indicate the 95% credible intervals for each term. Black dots indicate 95% credible intervals that did not include 0.

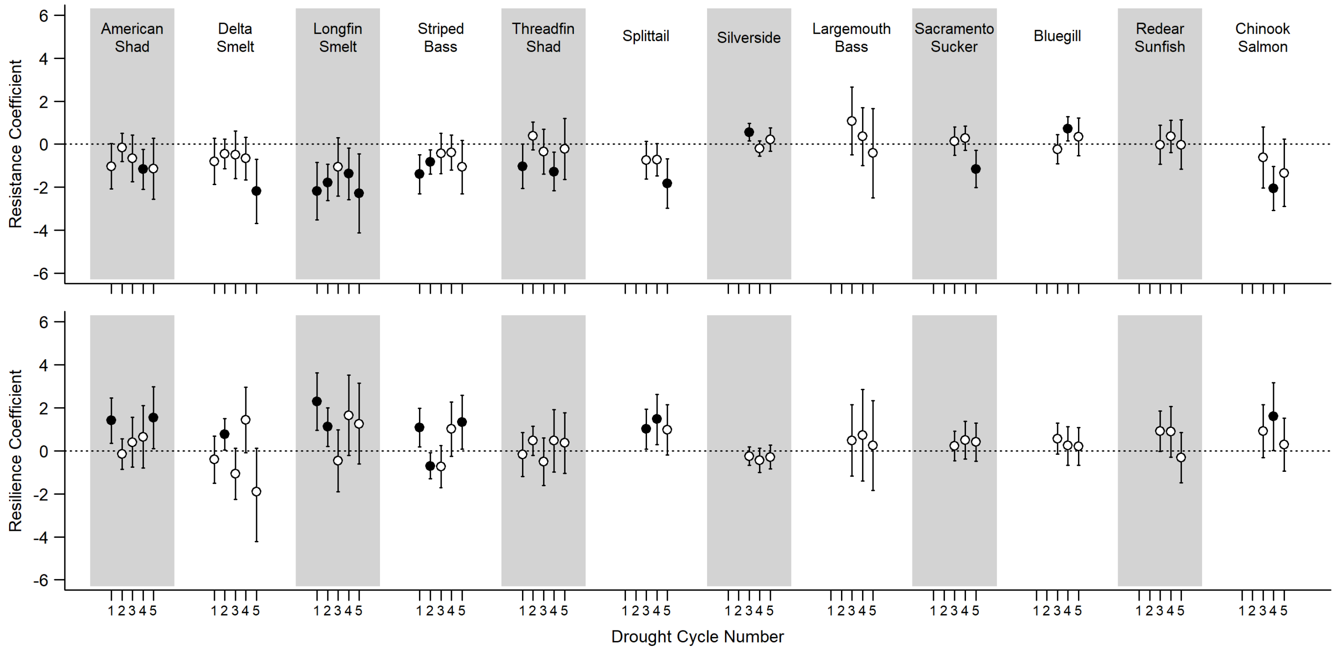

Table S1. List of FMWT stations, with + symbol indicating which stations have data in all three phases (pre-drought, drought, post-drought) of a drought cycle.

Station	Drought Cycle 1: 1967-1986	Drought Cycle 2: 1978-2000	Drought Cycle 3: 1995-2006	Drought Cycle 4: 2006-2011	Drought Cycle 5: 2011-2017
305	+	+	+	+	+
306	+	+	+	+	+
307	+	+	+	+	+
308	+	+	+	+	+
309	+	+	+	+	+
310	+	+	+	+	+
311	+	+	+	+	+
314	+	+	+	+	+
315	+	+	+	+	+
321	+	+	+	+	+
322	+	+	+	+	+
323	+	+	+	+	+
325	+	+	+	+	+
326	+	+	+	+	+
327	+	+	+	+	+
328	+	+	+	+	+
329	+	+	+	+	+
334	+	+	+	+	+
335	+	+	+	+	+
336	+	+	+	+	+
337	+	+	+	+	+
338	+	+	+	+	+
339	+	+	+	+	+
340	+	+	+	+	+
401	+	+	+	+	+
403	+	+	+	+	+
404	+	+	+	+	+
405	+	+	+	+	+
406	+	+	+	+	+
407	+	+	+	+	+
408	+	+	+	+	+
409	+	+	+	+	+
410	+	+	+	+	+
411	+	+	+	+	+
412	+	+	+	+	+
413	+	+	+	+	+

414	+	+	+	+	+
415	+	+	+	+	+
416	+	+	+	+	+
417	+	+	+	+	+
418	+	+	+	+	+
501	+	+	+	+	+
502	+	+	+	+	+
503	+	+	+	+	+
504	+	+	+	+	+
505	+	+	+	+	+
507	+	+	+	+	+
508	+	+	+	+	+
509	+	+	+	+	+
510	+	+	+	+	+
511	+	+	+	+	+
512	+	+	+	+	+
513	+	+	+	+	+
515	+	+	+	+	+
516	+	+	+	+	+
517	+	+	+	+	+
518	+	+	+	+	+
519	+	+	+	+	+
601	+	+	+	+	+
602	+	+	+	+	+
603	+	+	+	+	+
604	+	+	+	+	+
605	+	+	+	+	+
606	+	+	+	+	+
608	+	+	+	+	+
701	+	+	+	+	+
703	+	+	+	+	+
704	+	+	+	+	+
705	+	+	+	+	+
706	+	+	+	+	+
707	+	+	+	+	+
708	+	+	+	+	+
709	+	+	+	+	+
710	+	+	+	+	+
711	+	+	+	+	+
802	+	+	+	+	+
804	+	+	+	+	+
806	+	+	+	+	+
807	+	+	+	+	+

808	+	+	+	+	+
809	+	+	+	+	+
810	+	+	+	+	+
811	+	+	+	+	+
812	+	+	+	+	+
813	+	+	+	+	+
814	+	+	+	+	+
815	+	+	+	+	+
902	+	+	+	+	+
903	+	+	+	+	+
904	+	+	+	+	+
905	+	+	+	+	+
906	+	+	+	+	+
908	+	+	+	+	+
909	+	+	+	+	+
910	+	+	+	+	+
911	+	+	+	+	+
912	+	+	+	+	+
913	+	+	+	+	+
914	+	+	+	+	+
915	+	+	+	+	+
712	-	-	+	+	+
713	-	-	+	+	+
715	-	-	+	+	+
716	-	-	+	+	+
717	-	-	+	+	+
719	-	-	-	+	+
721	-	-	-	+	+
723	-	-	-	+	+
795	-	-	-	+	+
796	-	-	-	+	+
797	-	-	-	+	+

Table S2. List of DJFMP stations, with + symbol indicating which stations have data in all three phases (pre-drought, drought, post-drought) of a drought cycle. *Note that for Chinook Salmon, data for drought cycle 3 only consists of years from 1999 to 2006.

Station	Region	Drought Cycle 3: 1995-2006*	Drought Cycle 4: 2006-2011	Drought Cycle 5: 2011-2017	Notes
SR071E	1	+	+	+	
SR080E	1	+	+	+	
SR090W	1	+	+	+	
SR094E	1	+	+	+	
SR130E	1	+	+	+	
SR138E	1	+	+	+	
SR144W	1	+	+	+	
MS001N	2	+	+	+	
AM001S	2	+	+	+	
SR049E	2	+	+	+	
SR060E	2	+	+	+	
SR012E/SR012W	2	+	+	+	Station SR012E was replaced by SR012W, treated as replicates of one another.
SR014W	2	+	+	+	
SR017E	2	+	+	+	
SR024E	2	+	+	+	
SR043W	2	+	+	+	
SS011N	2	+	+	+	
LP003E	3	+	+	+	
MK004W	3	+	+	+	
SJ001S	3	+	+	+	
SJ005N	3	+	+	+	
TM001N	3	+	+	+	
DS002S	3	+	+	+	
SF014E	3	+	+	+	
GS010E	3	+	+	+	
XC001N	3	+	+	+	
SJ051E	4	+	+	+	
MR010W	4	+	+	+	
SJ026S	4	-	+	+	
SJ032S	4	+	+	+	
SJ041N	4	+	+	+	
OR003W	4	+	+	+	
OR014W	4	+	+	+	
OR019E	4	+	+	+	
WD002W	4	+	+	+	

OR023E	4	+	+	+	
SJ068W	5	+	+	-	
SJ056E	5	+	+	+	
SJ058W	5	+	+	+	
SJ063W	5	+	+	+	
SJ074W	5	+	+	+	
SJ077E	5	+	+	+	
SJ083W	5	+	+	+	

Table S3: Summary of Bayesian logistic regression model parameters and simulation accuracy rates for (a) American Shad, (b) Delta Smelt, (c) Longfin Smelt, (d) Striped Bass, (e) Threadfin Shad, (f) Sacramento Splittail, (g) Mississippi Silverside, (h) Largemouth Bass, (i) Sacramento Sucker, (j) Bluegill, (k) Redear Sunfish, and (l) Chinook Salmon. Columns in parameter summaries represent the marginal posterior mean (Mean), standard deviation (SD), and 95% credible interval lower (Lower CI) and upper (Upper CI) bounds. Simulated observation accuracy rates are summarized below parameter summaries. Columns in accuracy rate summaries represent the minimum (Min), first quartile (Q1), median (Median), mean (Mean), third quartile (Q3), and maximum (Max) accuracy rates. Potential scale reduction factors across all parameters and random effects, across all models, range from 0.999 to 1.003.

(a) American Shad

Parameter	Mean	SD	Lower CI	Upper CI
α	0.325017	0.325537	-0.31731	0.958914
$\Delta_{1,c=1}$	-1.03025	0.536392	-2.07922	0.032679
$\Delta_{2,c=1}$	1.409269	0.53778	0.355369	2.461786
$\Delta_{1,c=2}$	-0.15456	0.335083	-0.81298	0.49977
$\Delta_{2,c=2}$	-0.14272	0.359575	-0.84967	0.563881
$\Delta_{1,c=3}$	-0.65233	0.551611	-1.75215	0.433164
$\Delta_{2,c=3}$	0.391413	0.586456	-0.76534	1.556605
$\Delta_{1,c=4}$	-1.16907	0.471626	-2.1012	-0.24481
$\Delta_{2,c=4}$	0.648017	0.734574	-0.79451	2.093257
$\Delta_{1,c=5}$	-1.14281	0.721002	-2.56705	0.285985
$\Delta_{2,c=5}$	1.535939	0.731451	0.106451	2.986957
σ_m	0.347845	0.238769	0.125966	0.976459
σ_r	0.659873	0.080799	0.52261	0.840296
σ_s	0.761755	0.056718	0.659184	0.881065

Accuracy rate summary:

Min	Q1	Median	Mean	Q3	Max
0.028	0.459	0.638	0.610	0.775	0.989

(b) Delta Smelt

Parameter	Mean	SD	Lower CI	Upper CI
α	-1.41887	0.350193	-2.11046	-0.72774
$\Delta_{1,c=1}$	-0.80099	0.548643	-1.8773	0.287588
$\Delta_{2,c=1}$	-0.3932	0.553576	-1.50772	0.694446
$\Delta_{1,c=2}$	-0.45096	0.349645	-1.14309	0.243778
$\Delta_{2,c=2}$	0.763025	0.372485	0.029708	1.496553
$\Delta_{1,c=3}$	-0.48727	0.560279	-1.60563	0.614925
$\Delta_{2,c=3}$	-1.07632	0.602088	-2.26097	0.114563
$\Delta_{1,c=4}$	-0.66729	0.502768	-1.65835	0.320025
$\Delta_{2,c=4}$	1.444691	0.772586	-0.08502	2.964449
$\Delta_{1,c=5}$	-2.18566	0.764279	-3.68731	-0.69057

$\Delta_{2,c=5}$	-1.89614	1.09477	-4.21	0.123807
σ_m	0.239646	0.180187	0.070539	0.711816
σ_r	0.666319	0.085238	0.5211	0.853763
σ_s	2.170588	0.181523	1.845605	2.552485

Accuracy rate summary:

Min	Q1	Median	Mean	Q3	Max
0.003	0.766	0.953	0.833	0.992	1.000

(c) Longfin Smelt

Parameter	Mean	SD	Lower CI	Upper CI
α	-0.49989	0.712441	-1.95344	0.871917
$\Delta_{1,c=1}$	-2.18621	0.677994	-3.51985	-0.85051
$\Delta_{2,c=1}$	2.292388	0.678046	0.959668	3.62474
$\Delta_{1,c=2}$	-1.79319	0.428233	-2.62774	-0.93439
$\Delta_{2,c=2}$	1.11643	0.456152	0.20895	2.004925
$\Delta_{1,c=3}$	-1.05572	0.691438	-2.42144	0.301382
$\Delta_{2,c=3}$	-0.46866	0.727923	-1.8926	0.97061
$\Delta_{1,c=4}$	-1.37775	0.605789	-2.58467	-0.18701
$\Delta_{2,c=4}$	1.633978	0.953206	-0.21522	3.520598
$\Delta_{1,c=5}$	-2.28713	0.935881	-4.12501	-0.44696
$\Delta_{2,c=5}$	1.250737	0.951335	-0.61257	3.150086
σ_m	1.092857	0.592423	0.489391	2.579697
σ_r	0.833356	0.104518	0.656928	1.06439
σ_s	1.827413	0.143559	1.572844	2.136054

Accuracy rate summary:

Min	Q1	Median	Mean	Q3	Max
0.002	0.641	0.864	0.770	0.972	1.000

(d) Striped Bass

Parameter	Mean	SD	Lower CI	Upper CI
α	0.596779	0.266005	0.081007	1.125655
$\Delta_{1,c=1}$	-1.39652	0.45853	-2.30336	-0.49643
$\Delta_{2,c=1}$	1.083626	0.457162	0.184152	1.974991
$\Delta_{1,c=2}$	-0.83017	0.285779	-1.39345	-0.26607
$\Delta_{2,c=2}$	-0.7035	0.308402	-1.30416	-0.09292
$\Delta_{1,c=3}$	-0.43638	0.470188	-1.36456	0.500125
$\Delta_{2,c=3}$	-0.74411	0.496092	-1.71928	0.244879
$\Delta_{1,c=4}$	-0.39312	0.412038	-1.20643	0.420893
$\Delta_{2,c=4}$	1.015868	0.64128	-0.25636	2.277012
$\Delta_{1,c=5}$	-1.06502	0.628059	-2.29876	0.165852
$\Delta_{2,c=5}$	1.335681	0.631964	0.088366	2.578507
σ_m	0.205225	0.160282	0.061053	0.630479
σ_r	0.554743	0.069635	0.436559	0.710169

σ_s	1.220561	0.089129	1.058664	1.410115

Accuracy rate summary:

Min	Q1	Median	Mean	Q3	Max
0.011	0.556	0.787	0.712	0.913	1.000

(e) Threadfin Shad

Parameter	Mean	SD	Lower CI	Upper CI
α	-0.14986	0.536359	-1.19244	0.878876
$\Delta_{1,c=1}$	-1.03228	0.518964	-2.04805	-0.00152
$\Delta_{2,c=1}$	-0.16288	0.521132	-1.18618	0.853645
$\Delta_{1,c=2}$	0.376541	0.327032	-0.26725	1.026107
$\Delta_{2,c=2}$	0.466713	0.346766	-0.21344	1.151065
$\Delta_{1,c=3}$	-0.34405	0.528768	-1.38965	0.691533
$\Delta_{2,c=3}$	-0.49969	0.559089	-1.60282	0.598116
$\Delta_{1,c=4}$	-1.27807	0.457077	-2.17153	-0.37303
$\Delta_{2,c=4}$	0.485377	0.734264	-0.97475	1.924319
$\Delta_{1,c=5}$	-0.22832	0.716357	-1.64288	1.194749
$\Delta_{2,c=5}$	0.369029	0.713905	-1.05404	1.771046
σ_m	0.795161	0.461148	0.33793	1.942431
σ_r	0.62894	0.078425	0.496746	0.802046
σ_s	1.521028	0.111642	1.320218	1.755508

Accuracy rate summary:

Min	Q1	Median	Mean	Q3	Max
0.013	0.578	0.812	0.726	0.930	1.000

(f) Sacramento Splittail

Parameter	Mean	SD	Lower CI	Upper CI
α	-2.29816	0.941304	-4.18754	-0.39098
$\Delta_{1,c=3}$	-0.74964	0.437952	-1.61865	0.132387
$\Delta_{2,c=3}$	1.017388	0.464874	0.086346	1.939517
$\Delta_{1,c=4}$	-0.72018	0.377046	-1.4669	0.030199
$\Delta_{2,c=4}$	1.469639	0.589328	0.289579	2.622284
$\Delta_{1,c=5}$	-1.82758	0.578041	-2.96642	-0.67265
$\Delta_{2,c=5}$	0.973678	0.585645	-0.18455	2.134355
σ_m	2.099887	0.766865	1.168846	4.0517
σ_r	0.511953	0.103219	0.351701	0.752326
σ_s	0.849389	0.100449	0.678323	1.071716

Accuracy rate summary:

Min	Q1	Median	Mean	Q3	Max
0.003	0.662	0.904	0.790	0.982	1.000

(g) Mississippi Silverside

Parameter	Mean	SD	Lower CI	Upper CI
α	-0.30091	0.48973	-1.24428	0.68406
$\Delta_{1,c=3}$	0.558685	0.202943	0.15838	0.966614
$\Delta_{2,c=3}$	-0.25078	0.215936	-0.68018	0.177816
$\Delta_{1,c=4}$	-0.19713	0.177515	-0.5477	0.154213
$\Delta_{2,c=4}$	-0.44223	0.279621	-1.00296	0.119259
$\Delta_{1,c=5}$	0.216108	0.27368	-0.32579	0.755824
$\Delta_{2,c=5}$	-0.28702	0.278798	-0.83694	0.267844
σ_m	0.995405	0.385186	0.539746	1.933433
σ_r	0.23022	0.050356	0.151009	0.345729
σ_s	1.300186	0.145548	1.050652	1.619404

Accuracy rate summary:

Min	Q1	Median	Mean	Q3	Max
0.010	0.496	0.699	0.656	0.852	0.992

(h) Largemouth Bass

Parameter	Mean	SD	Lower CI	Upper CI
α	-3.45139	0.610931	-4.68455	-2.24716
$\Delta_{1,c=3}$	1.073656	0.792971	-0.49961	2.658267
$\Delta_{2,c=3}$	0.474855	0.841549	-1.18199	2.153687
$\Delta_{1,c=4}$	0.357826	0.678304	-0.99291	1.690563
$\Delta_{2,c=4}$	0.724206	1.073636	-1.40315	2.860892
$\Delta_{1,c=5}$	-0.41142	1.048883	-2.49447	1.664063
$\Delta_{2,c=5}$	0.250561	1.054283	-1.84138	2.334447
σ_m	0.940282	0.35937	0.505983	1.859247
σ_r	0.94336	0.194673	0.640181	1.400696
σ_s	1.270491	0.149554	1.014578	1.599744

Accuracy rate summary:

Min	Q1	Median	Mean	Q3	Max
0.002	0.621	0.858	0.760	0.959	1.000

(i) Sacramento Sucker

Parameter	Mean	SD	Lower CI	Upper CI
α	-2.47319	0.641526	-3.74442	-1.19832
$\Delta_{1,c=3}$	0.135526	0.330017	-0.52096	0.798621
$\Delta_{2,c=3}$	0.224993	0.346002	-0.45723	0.906164
$\Delta_{1,c=4}$	0.27278	0.284493	-0.28526	0.83278
$\Delta_{2,c=4}$	0.500238	0.443757	-0.3826	1.379198
$\Delta_{1,c=5}$	-1.15176	0.437598	-2.01538	-0.27756
$\Delta_{2,c=5}$	0.412424	0.446874	-0.47825	1.292816
σ_m	1.118729	0.423386	0.609799	2.191103
σ_r	0.380917	0.079026	0.258854	0.564739
σ_s	2.441103	0.308086	1.91458	3.128333

Accuracy rate summary:

Min	Q1	Median	Mean	Q3	Max
0.005	0.582	0.828	0.749	0.971	1.000

(j) Bluegill

Parameter	Mean	SD	Lower CI	Upper CI
α	-3.48222	0.304925	-4.09595	-2.88367
$\Delta_{1,c=3}$	-0.24073	0.346368	-0.91764	0.447445
$\Delta_{2,c=3}$	0.560391	0.365155	-0.15878	1.295499
$\Delta_{1,c=4}$	0.715977	0.286302	0.144434	1.284408
$\Delta_{2,c=4}$	0.23673	0.450311	-0.6716	1.124549
$\Delta_{1,c=5}$	0.334311	0.438723	-0.52502	1.210717
$\Delta_{2,c=5}$	0.204433	0.437193	-0.67315	1.078909
σ_m	0.17584	0.094596	0.065805	0.416541
σ_r	0.37719	0.087827	0.237454	0.57927
σ_s	1.519669	0.193453	1.193541	1.951425

Accuracy rate summary:

Min	Q1	Median	Mean	Q3	Max
0.003	0.838	0.938	0.844	0.980	1.000

(k) Redear Sunfish

Parameter	Mean	SD	Lower CI	Upper CI
α	-4.24516	0.415116	-5.06468	-3.44194
$\Delta_{1,c=3}$	-0.02302	0.451675	-0.9245	0.875869
$\Delta_{2,c=3}$	0.921322	0.472009	-0.01686	1.854386
$\Delta_{1,c=4}$	0.364545	0.379224	-0.39502	1.121381
$\Delta_{2,c=4}$	0.883665	0.596385	-0.30217	2.064014
$\Delta_{1,c=5}$	-0.02615	0.581384	-1.17015	1.129934
$\Delta_{2,c=5}$	-0.31878	0.585642	-1.48568	0.846257
σ_m	0.446131	0.193557	0.218583	0.929301
σ_r	0.509242	0.107776	0.340305	0.761519
σ_s	1.821438	0.229317	1.432721	2.327386

Accuracy rate summary:

Min	Q1	Median	Mean	Q3	Max
0.001	0.860	0.963	0.866	0.991	1.000

(l) Chinook Salmon

Parameter	Mean	SD	Lower CI	Upper CI
α	-1.07827	1.000967	-3.04301	0.877381
$\Delta_{1,c=3}$	-0.6247	0.719438	-2.0434	0.809405
$\Delta_{2,c=3}$	0.916806	0.619422	-0.31497	2.145336
$\Delta_{1,c=4}$	-2.05282	0.512618	-3.07346	-1.03507

$\Delta_{2,c=4}$	1.603255	0.794203	0.023621	3.170159
$\Delta_{1,c=5}$	-1.34532	0.779127	-2.89213	0.231711
$\Delta_{2,c=5}$	0.290139	0.619123	-0.95287	1.508615
σ_m	1.885809	0.682384	1.050548	3.623147
σ_r	0.699313	0.155273	0.465339	1.070536
σ_s	1.894344	0.224001	1.511985	2.390495

Accuracy rate summary:

Min	Q1	Median	Mean	Q3	Max
0.004	0.614	0.853	0.758	0.961	1.000
