# Supplementary material for: Resistance and resilience of pelagic and littoral fishes to drought in the San Francisco Estuary
Source: Ecol Appl. 2021 Jan 22;31(2):e02243. doi: 10.1002/eap.2243 (PMC7988542; doi:10.1002/eap.2243)
Supplement: Supplementary file 3 — Metadta S1 [file EAP-31-e02243-s001.pdf]

Mahardja, B., V. Tobias, S. Khanna, L. Mitchell, P. Lehman, T. Sommer, L. Brown, S. Culberson, and J.L. Conrad. 2020. Resistance and resilience of pelagic and littoral fishes to drought in the San Francisco Estuary. Ecological Applications.

Data S1

R code to format data sets, run ANOVA, as well as the drought resistance and resiliency models for all twelve species as described in the manuscript. Full data sets can be found in the following data repository website: https://doi.org/10.6084/m9.figshare.12855581.v3

Authors

Brian Mahardja
United States Fish and Wildlife Service
850 S. Guild Ave., Lodi, CA, USA
brian_mahardja@fws.gov
ORCID: 0000-0003-0695-3745

Lara Mitchell
United States Fish and Wildlife Service
850 S. Guild Ave., Lodi, CA, USA
lara_mitchell@fws.gov

Vanessa Tobias
United States Fish and Wildlife Service
850 S. Guild Ave., Lodi, CA, USA
vanessa_tobias@fws.gov

File list (files found within DataS1.zip)

`Mahardja_et_al_2020_Drought_Resiliency.R`
`Mahardja_et_al_2020_Drought_ANOVA.R`

Description

`Mahardja_et_al_2020_Drought_Resiliency.R` – R code to format data sets,

run the drought resistance and resiliency models, and produce figures for all 12 fish species as described in the manuscript.

`Mahardja_et_al_2020_Drought_ANOVA.R` – R code to conduct ANOVA tests on several hydrologic variables as seen in Table 1 of the manuscript. This was used to ensure the validity of the drought and wet period classifications.
